# Evaluation of Nitinol Staples for the Lapidus Arthrodesis in a Reproducible Biomechanical Model

**DOI:** 10.3389/fsurg.2015.00065

**Published:** 2015-12-14

**Authors:** Nicholas A. Russell, Gianmarco Regazzola, Amiethab Aiyer, Tomohiro Nomura, Matthew H. Pelletier, Mark Myerson, William R. Walsh

**Affiliations:** ^1^Surgical and Orthopaedic Research Laboratories, Prince of Wales Clinical School, University of New South Wales, Sydney, NSW, Australia; ^2^Institute for Foot and Ankle, Mercy Medical Centre, Baltimore, MD, USA

**Keywords:** nitinol staple, Lapidus model, osteotomy, shape memory, biomechanical

## Abstract

While the Lapidus procedure is a widely accepted technique for treatment of hallux valgus, the optimal fixation method to maintain joint stability remains controversial. The purpose of this study is to evaluate the biomechanical properties of new shape memory alloy (SMA) staples arranged in different configurations in a repeatable first tarsometatarsal arthrodesis model. Ten sawbones models of the whole foot (*n* = 5 per group) were reconstructed using a single dorsal staple or two staples in a delta configuration. Each construct was mechanically tested non-destructively in dorsal four-point bending, medial four-point bending, dorsal three-point bending, and plantar cantilever bending with the staples activated at 37°C. The peak load (newton), stiffness (newton per millimeter), and plantar gapping (millimeter) were determined for each test. Pressure sensors were used to measure the contact force and area of the joint footprint in each group. There was a statistically significant increase in peak load in the two staple constructs compared to the single staple constructs for all testing modalities with *P* values range from 0.016 to 0.000. Stiffness also increased significantly in all tests except dorsal four-point bending. Pressure sensor readings showed a significantly higher contact force at time zero (*P* = 0.037) and contact area following loading in the two staple constructs (*P* = 0.045). Both groups completely recovered any plantar gapping following unloading and restored their initial contact footprint. The biomechanical integrity and repeatability of the models was demonstrated with no construct failures due to hardware or model breakdown. SMA staples provide fixation with the ability to dynamically apply and maintain compression across a simulated arthrodesis following a range of loading conditions.

## Introduction

The Lapidus procedure and its modifications include an arthrodesis of the first metatarsal cuneiform joint introduced in 1934 (1). The procedure was originally described for the treatment of patients with metatarsus primus varus; however, it has since been used with clinical success for addressing hallux valgus deformities, arthritis, for adolescent bunions, hypermobility of the first ray, and in the revision setting (2–5). Crossed-screw repair is currently the most widely used fixation technique; however, complication rates and non-union have been reported clinically in 5–15% of cases (6–8). Other techniques using plates, mechanical staples, K-wires, pins, and various combinations and configurations of each have also been used with varying success (4, 5, 9). An ideal fixation technique would provide mechanical stability at the arthrodesis, minimize micro motion, and apply adequate compression to maintain contact area and pressure at the interface to facilitate fusion (10, 11). None of the current hardware options available to surgeons are able to fulfill these requirements.

Shape memory alloy (SMA) staples are part of a broader category of metallic compression staples, which have been used clinically for fracture fixation, arthrodesis, and osteotomies (12). SMA staples are made from Nitinol, a near equiatomic alloy of nickel and titanium, which exhibits unique superelastic and shape memory characteristics. These properties are brought about by a reversible solid–solid phase transformation from a highly ordered austenitic crystal structure to a less ordered martensitic structure (13, 14). SMA staples exploit the properties of Nitinol in a narrow temperature range above its transformation temperature where it possesses superelasticity and exists in its stronger austenitic state. This temperature range includes body temperature (15, 16), allowing SMA staples to be implanted into the body where they undergo a thermally induced phase transformation and conformation change in shape. Due to their superelastic and shape memory characteristics, SMA staples have the ability elastically recover from large deformations, which may occur *in vivo*, imparting a dynamic compressive capability not possible in conventional mechanical staples. This has been demonstrated in a number of *in vitro* biomechanical studies which have reported that SMA staples generate a greater compression across a simulated osteotomy compared to mechanical staples (11, 17), and resist permanent deformation, fully recovering their shape following loading (18).

Clinically, the properties of SMA staples offer many potential benefits for the Lapidus arthrodesis. The ability to apply and maintain a uniform compression across an arthrodesis should yield more primary bone healing as there is an increased resistance to mechanical stresses and micromotion during the healing period. Furthermore, unlike screw fixation that occupies valuable area across the joint interface, staples allow full joint coaptation, maximizing the joint footprint for fusion. Despite this, clinical research evaluating the use of SMA staples for the Lapidus arthrodesis is scant. In the only study, to date, Mallette et al. (19) evaluated the non-union rates of two SMA staples, the EZ Clip (MMI, TN, USA) and OS Staple (BME, TX, USA), utilized in 36 first TMT arthrodeses. They reported non-union in 8.3% of cases, which is well within the range of what has been reported for other forms of fixation (4, 6–8, 20).

Despite this anecdotal evidence on the merits of these new staples, there is a no scientific data in a reproducible model supporting these assertions. Consequently, this study aims to evaluate the biomechanical performance of two new SMA staples deployed independently or in a delta configuration in a first TMT arthrodesis model. The constructs were tested using different loading modalities to simulate the broad mechanical loading conditions experienced during gait and to assess the robustness of the model.

## Materials and Methods

### Construct Preparation

Sawbones full foot models (#1131, Pacific Research Laboratories, WA, USA) of the left foot were used in this study. These solid polyurethane foam models are identical and have a uniform density and compressive modulus (250.2 ± 41.6 MPa) and provide an anatomically relevant comparison. Ten models were randomly assigned to one of two treatment groups (*n* = 5 per group) (Table 1). A band saw was used to section each model to isolate the first TMT and allow for consistent placement on the mechanical testing jig. Each model was then marked out with a line down the mid-axis of the cuneiform and the first metatarsal, two dots just below the line on the cuneiform and the metatarsal and two dots on the nail plate. These markings were made in order to establish appropriate alignment in the sagittal, coronal, and transverse planes.

**Table 1:** **Summary of the study groups and implant configuration**.

Test group	Sample size	Implants	Configuration
1	5	Single BME SPEED staple (SE-2020TI, *BioMedical Enterprises*, San Antonio, TX, USA)	Dorsal
2	5	Two BME SPEED staples (SE-2020, *BioMedical Enterprises*, San Antonio, TX, USA)	One dorsal, one medial and slightly plantar (delta configuration)

**Figure 1:**
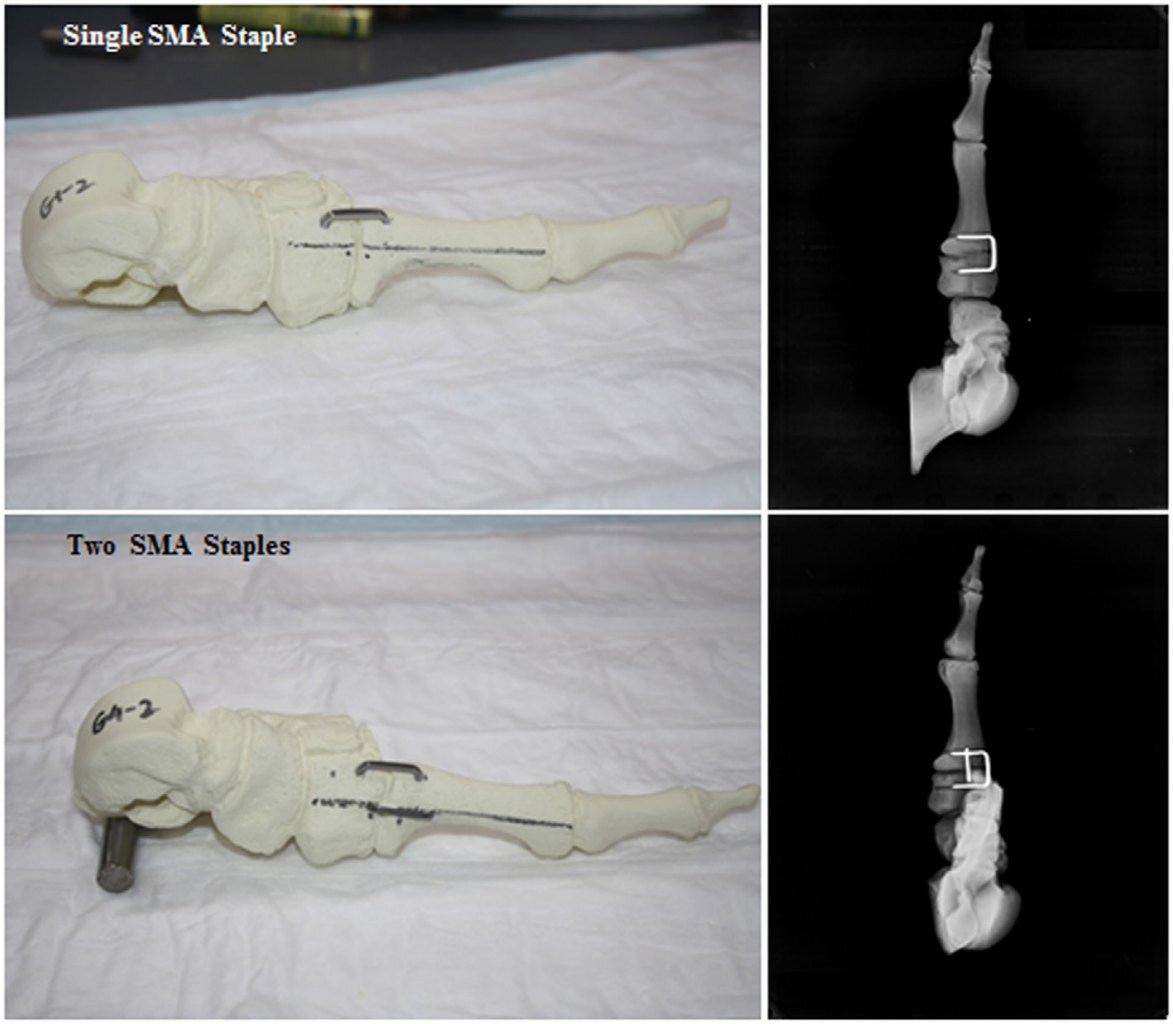
**Digital photographs and faxitron radiographs showing the assembled single SMA staple and double SMA staple constructs**.

In both groups, the dorsal SMA staples (*Speed*, BME, San Antonio, TX, USA) were placed approximately 5 mm from the intercuneiform/intermetatarsal joints. Given the dorsal staples were 20 mm in length, a ruler was used to ensure that the legs of the staple were 10 mm from the joint surface. A drill guide and 2.65 mm drill were used to create the holes for the staples. An oscillating saw was used to detach the first metatarsal from the cuneiform. Sandpaper (120 grit) was use to smooth down the edges of the cut. Using the alignment markings, the metatarsal was held in a reduced position against the cuneiform. The staple was then inserted and disengaged from the deployment device. In the double staple group, the drill guide was offset 5 mm proximally to prevent interference with the legs of the first staple and to accommodate the second medial staple. Faxitron radiographs were taken of each construct to confirm correct staple placement (Figure 1).

### Contact Force and Area Measurement

Prior to mechanical testing, a calibrated pressure sensor (model 4000, *TekScan*, MA, USA) was placed between the joint surfaces of each construct and samples were equilibrated at 37°C in a temperature controlled incubator. A time zero reading was taken for calculation of the initial contact force and area. The constructs were subsequently tested mechanically, with another measurement taken following the dorsal four-point bending test to assess the ability of the staples to restore the contact footprint.

### Mechanical Testing

The constructs were mechanically tested using a servo-hydraulic testing machine (MTS Bionix, MN, USA) in dorsal four-point bending, medial four-point bending, dorsal three-point bending, and plantar cantilever bending. The constructs were tested to 3 mm axial displacement at 1 mm/min in the three- and four-point bending tests and to 25 mm axial displacement at 10 mm/min in the cantilever bending tests. A hair dryer was used to provide a constant heat source to ensure the SMA staples remained activated during each test to 37°C. Temperature was confirmed using an infrared thermal imaging camera (thermoIMAGER TIM 160, Micro-Epsilon, Germany). Peak load and stiffness were calculated from the load–displacement output of each test.

### Plantar Gapping

Digital photographs of the osteotomy were synchronous with the mechanical testing and taken every 6 s for the duration. Plantar gapping was then evaluated by measuring the distance between the distal edges of the joint interface using an in-house Matlab subroutine (Matlab R2014a, *MathWorks*, MA, USA). Gapping values were determined prior to loading, at 1, 2, and 3 mm of actuator displacement and following unloading to determine the recovery of each construct. Additionally, gapping results were correlated with the load output to a give load versus gapping plot. Representative images of the plantar gapping experienced by both groups during each testing modality are shown in Figure 5.

### Statistical Analysis

An analysis of variance was used to compare all mechanical testing outcomes between the two groups using IBM SPSS Version 22. Similarly, changes in the contact force and contact area following mechanical testing were compared both within and between groups. Any difference with the *P* value <0.05 was considered to be significant.

## Results

**Figure 2:**
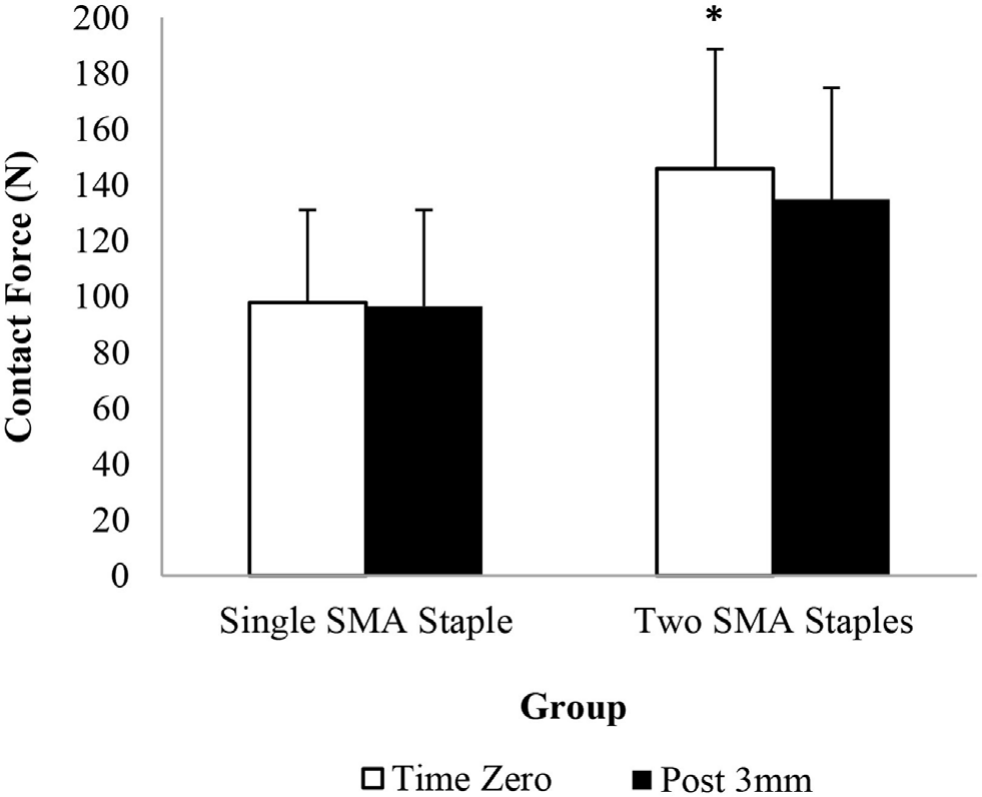
Interfragmentary contact force of each group at time zero and following 3 mm of dorsal four-point bending; * denotes a statistically significant increase at *P* < 0.05 compared to the single SMA staple group.

**Figure 3:**
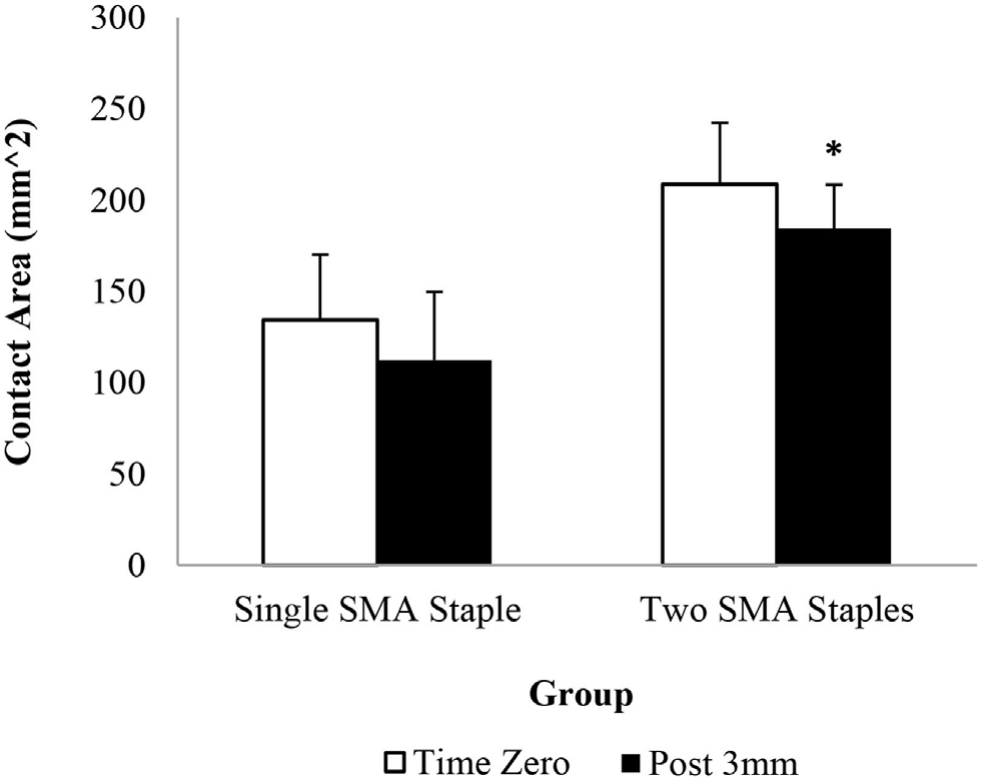
Interfragmentary contact area of each group at time zero and following 3 mm of dorsal four-point bending; * denotes a statistically significant increase at *P* < 0.05 compared to the single SMA staple group.

The pressure sensor results showed that the addition of the second SMA staple increased contact force and contact area compared to a single SMA staple alone at all readings (Figures 2 and 3). At time zero contact force and contact area increased by 48 and 55%, respectively, though only contact force was statistically significant (*P* = 0.037). Similarly, following loading there was a significant 64% (*P* = 0.045) increase in contact area in the two SMA staple constructs. Both staple groups maintained the time zero properties at the fusion interface, with no significant change observed in contact force or contact area following mechanical loading.

**Figure 4:**
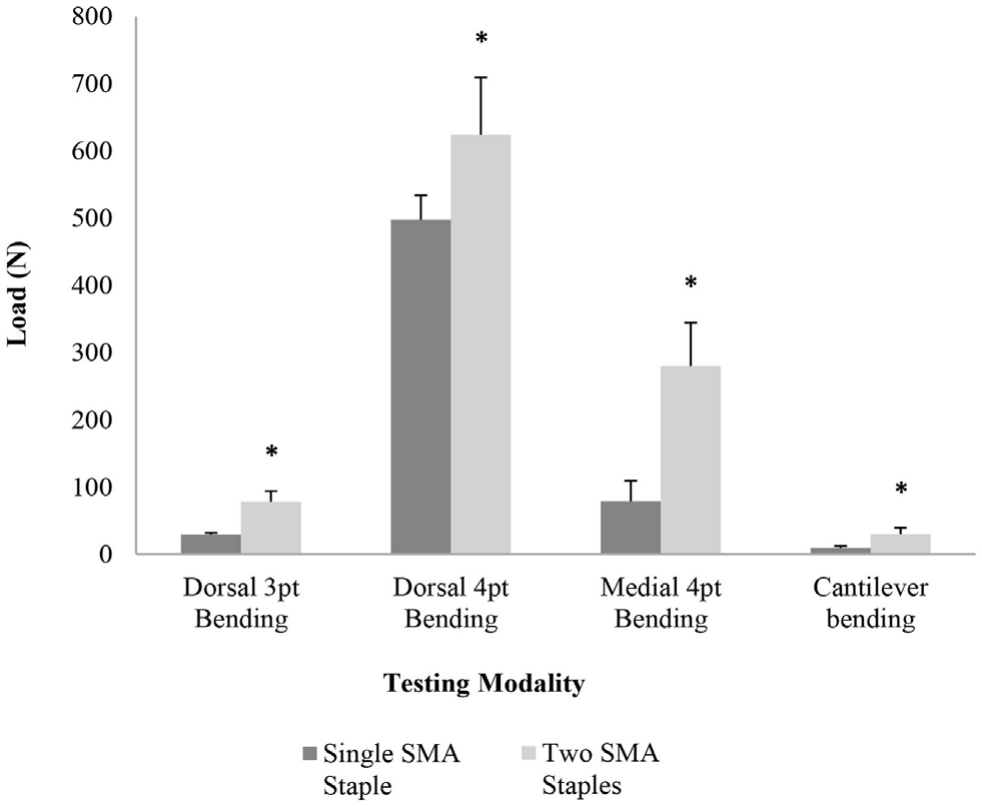
Mean peak load for each group in dorsal three-point bending, dorsal four-point bending, medial four-point bending, and cantilever bending; * denotes a statistically significant increase at *P* < 0.05 compared to the single SMA staple group.

The mechanical results showed a significant increase (*P* < 0.05) in peak load (Figure 4) for the two SMA staple constructs compared to the single SMA staple constructs for all testing modalities. The largest increase was found in medial four-point bending where there was a threefold increase in the peak load and a fourfold increase in energy. Stiffness increased significantly in the two SMA staple groups compared to the single SMA staple group for all testing modalities except dorsal four-point bending, where a similar stiffness of 250.7 and 217.1 N/mm was found, respectively (Table 2).

**Table 2:** **Summary of the mean and SD results for stiffness for each testing modality**.

Test group	Testing modality	Stiffness (N/mm)	

		Mean	SD
Single SMA staple	Dorsal 3pt bending	21.1* (12.4)	
	Dorsal 4pt bending	217.1 (37.2)	
	Medial 4pt bending	50.1* (26.4)	
	Cantilever bending	1.5* (0.5)	
Two SMA staples	Dorsal 3pt bending	64.9 (16.7)	
	Dorsal 4pt bending	250.7 (35.9)	
	Medial 4pt bending	169.9 (36.5)	
	Cantilever bending	3.8 (1.7)	
		

Values are mean (SD); * denotes a statistically significance reduction at *P* < 0.05 compared to the two staple group.

*Bold used to indicate significant differences*.

**Figure 5:**
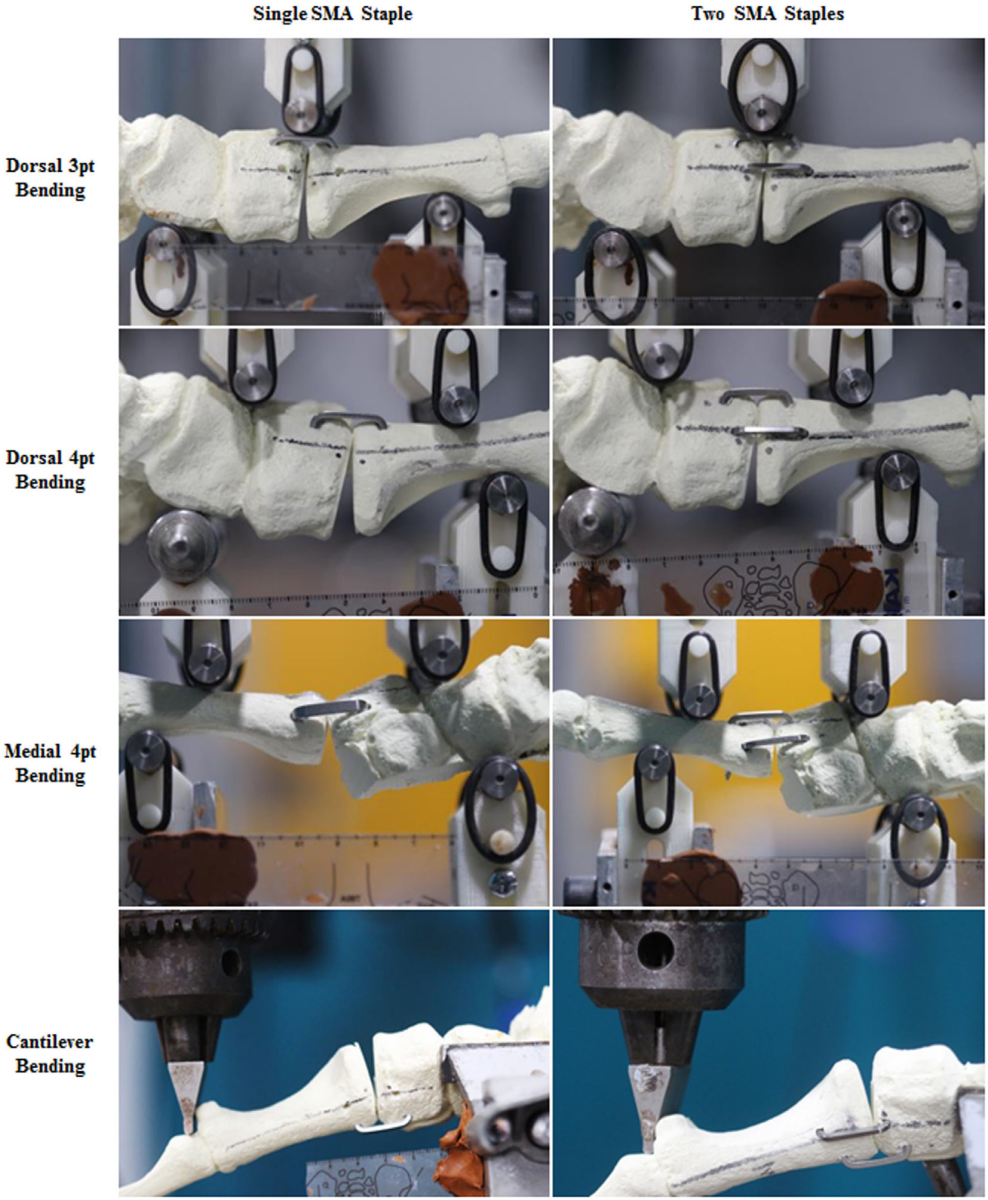
**Digital photograph graph plotting the load versus gapping results for each construct in dorsal four-point bending and medial four-point bending**.

Representative images of the plantar gapping experienced by both groups during each testing modality are shown in Figure 5. The gapping measurements showed that the addition of the dorso-medial staple had no significant effect on plantar gapping when loaded in dorsal three-point bending (Table 3). Conversely, when loaded dorsally in four-point bending there were statistically significant reductions in plantar gapping of 53% (*P* = 0.001), 43% (*P* = 0.002), and 41% (*P* = 0.002) in the two SMA staple constructs at 1, 2, and 3 mm of actuator displacement, respectively. Despite a significant increase in peak load and stiffness in the two SMA staple constructs during medial four-point bending, this did not translate to improved resistance to gapping with no statistical difference detected between groups. The use of the larger SMA staple in the single staple constructs reduced plantar gapping by 16% in cantilever bending, though this only approached statistical significance (*P* = 0.06). The dynamic nature of the staples was confirmed with a full recovery of any gapping in both constructs following all loading modalities.

**Table 3:** **Summary of the plantar gapping measurements for each testing modality at different actuator displacements**.

Test group	Testing modality	Plantar gapping (mm)				
		Bending			Cantilever	All tests
	
		1 mm	2 mm	3 mm	25 mm	Load removed
Single SMA staple	Dorsal 3pt bending	0.67 (0.18)	1.27 (0.51)	2.09 (0.48)	–	0.0 (0.0)
	Dorsal 4pt bending	1.01 (0.15)	2.65 (0.36)	4.71 (0.60)	–	0.0 (0.0)
	Medial 4pt bending	0.69 (0.47)	1.95 (0.39)	3.23 (0.59)	–	0.0 (0.0)
	Cantilever bending	–	–	–	5.04 (0.90)	0.0 (0.0)
Two SMA staples	Dorsal 3pt bending	0.51 (0.26)	1.21 (0.26)	1.97 (0.44)	–	0.0 (0.0)
	Dorsal 4pt bending	0.48* (0.19)	1.52* (0.40)	2.79* (0.75)	–	0.0 (0.0)
	Medial 4pt bending	0.23 (0.24)	1.50 (0.34)	2.83 (0.46)	–	0.0 (0.0)
	Cantilever bending	–	–	–	5.99 (0.33)	0.0 (0.0)

Values are mean (SD); * denotes a statistically significance reduction at *P* < 0.005 compared to the single SMA staple group.

*Bold used to indicate significant differences*.

**Figure 6:**
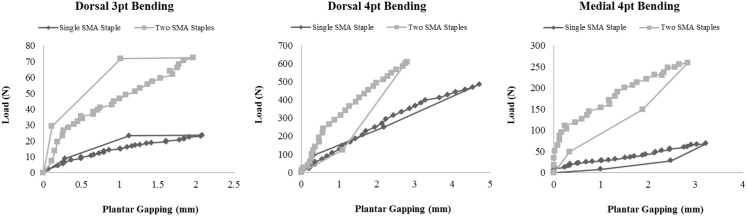
**Load versus gapping results for each construct in dorsal four-point bending and medial four-point bending**.

The load versus gapping plots highlight that the addition of the second dorso-medial staple increases the stiffness of the construct and limits plantar gapping to <3 mm in all mechanical loading scenarios (Figure 6).

## Discussion

The optimal reconstruction technique for the Lapidus arthrodesis remains controversial. Paramount to the success of any surgical approach is the ability to stabilize bony fragments, resist and recover plantar gapping and provide adequate compression across the fusion site (10, 21, 22). As a result, a large number of fixation techniques and devices have been utilized in order to obtain an ideal construct that could maximize rigidity, avoid excessive micromotion, and consequently reduce failure rates. SMA staples have unique properties, which offer many potential benefits for use in joint fusion. This study aims to evaluate the *in vitro* biomechanical performance of a new generation of SMA staples in a reproducible first TMT arthrodesis model. We specifically compared the differences in the biomechanical properties of a single dorsal SMA staple (*Speed*, BME, San Antonio, TX, USA) versus a double staple (dorsal and medial).

This study has presented a new biomechanical testing model for the Lapidus arthrodesis. Repaired constructs were mechanically tested under different loading modalities that apply different levels of bending and shear stress to the osteotomy. While it is impossible to fully replicate the loading experienced *in vivo*, the different modalities selected for this study aims to simulate some of the stresses experienced during gait. For instance, cantilever bending was used to model dorsiflexion during push-off. The sawbones samples used to provide a standardized anatomy, which controls for variations in size, bone density, loading, implant size, and allowed for accurate surgical placement of the fixation hardware. All these factors limit the scatter of the experimental data, and thus reduce experimental variation. Additionally, this allowed the contact force and area of the joint footprint to be calculated in a repeatable manner due to a consistent geometry. While the polyurethane foam does not replicate the mechanical properties of human bone, there was no evidence of hardware pull-out or cut through following multiple mechanical tests in different orientations. Given these factors, this testing model can provide a repeatable method of examining the various positive and negative aspects of different fixation methods for the Lapidus arthrodesis.

The results of this study highlighted the advantages of deploying two SMA staples compared to a single larger SMA staple for the Lapidus arthrodesis. There was an increased joint contact force and area at the osteotomy, a significant (*P* < 0.05) increase in peak load and a significant (*P* < 0.05) increase in the stiffness of the constructs for all loading modalities except dorsal four-point bending. These results reflect the role of the medial staple in imparting multi-planar rigidity and rotational stability to the construct. The plantar gapping results demonstrated the unique superelastic properties of the staples with a complete recovery and restoration of the joint footprint following unloading in each test. Moreover, the plantar gapping in all of the two staple bending tests was <3 mm, which has been used as a failure criterion in previous *in vitro* biomechanical studies (21–23).

While a number of other studies have evaluated the biomechanical properties of different fixation methods for the Lapidus arthrodesis (3, 5, 9, 17, 18, 21–28), variations in models and testing methods make meaningful comparison difficult. When comparing the rigidity of crossed-screw constructs with locking plates in a cadaveric study, Gruber et al. (21) reported peak loads in four-point bending of 110.8 and 120.4 N, respectively. There was no mention of the gage span used in their study making comparison of the moments impossible. In another cadaveric study, Scranton et al. (5) performed cantilever bending on plate and crossed-screw constructs using a similar technique to the present study. They reported failure moments of 6.0 and 4.4 Nm, respectively. The peak cantilever bending moment of the two SMA staple constructs in the present study was substantially lower (2.09 Nm), though there was a full recovery of all gapping following unloading. While these studies are not analogous, these results suggest that while SMA staple fixation is not as rigid as plate and crossed-screw fixation, their superelastic characteristics could be useful as an adjunctive means to provide dynamic compression and improve joint coaptation.

Other studies have demonstrated the compressive properties and recovery behavior of SMA staples in different models. Using a small load cell fitted between two perspex blocks, Farr et al. (17) reported a peak contact force of 35 N generated by a 20 × 20 *Memotech* SMA staple. A similar result was recorded by Shibuya et al. (11) using a pressure sensor in a sawbones calcaneus model. They reported a peak contact force of 32.65 N using a 20 × 20 *OSStaple*. While not directly comparable due to the different areas of contact and measurement methods, the single 20 × 20 TI *Speed* staple in the present study produced a contact force of 97.8 N. This drastic improvement is due to evolution in SMA staple design with a wider, low-profile bridge. These improvements are also evident from another study by Rentham et al. (18) comparing the mechanical performance of different staples in four-point bending. They reported a bending moment of 0.51 Nm in a 14 × 12 *Memoclip* staple at 10° of bending. Comparatively, a peak bending moment of 7.46 Nm was measured in the single SMA staple group in the present study. Again, these studies are not directly comparable due to the differences in staple size and model, but the results of these studies to demonstrate the improved rigidity and compressive performance of these new SMA staples.

This study is limited as it does not take the *in vivo* environment into account. The use of a synthetic anatomical model means that the role of soft tissue structures surrounding the joint are not considered. Furthermore, while a range of loading modalities were utilized; cyclic testing would be more indicative of *in vivo* loading conditions, and thus more clinically relevant. However, this model does provide investigators a means to reduce and control variables to a larger extent than with cadaveric models. Cadaver samples are not readily available, and the quality of the bone in the samples that are used is variable and prone to fracture and/or hardware pull-out.

This study has presented a reproducible biomechanical testing model for the Lapidus arthrodesis, which can provide a useful means to evaluate the various positive and negative aspects of different fixation constructs. The new generation of SMA staples has improved rigidity and compressive capabilities which when used alone or as an adjunctive hardware imparts dynamic fixation, which may improve clinical outcomes for the Lapidus arthrodesis compared to the existing techniques.

## Author Contributions

All authors (NR, GR, AA, TN, MP, MM, and WW) have contributed to substantial contributions to the conception or design of the work; or the acquisition, analysis, or interpretation of data for the work; drafting the work or revising it critically for important intellectual content; final approval of the version to be published; and agreement to be accountable for all aspects of the work in ensuring that questions related to the accuracy or integrity of any part of the work are appropriately investigated and resolved.

## Conflict of Interest Statement

Sawbone models and implants were provided by Biomedical Enterprises (BME, San Antonio, TX, USA).
